# Photopolymerization Parameters Influence Mechanical, Microstructural, and Cell Loading Properties of Rapidly Fabricated Cell Scaffolds

**DOI:** 10.1021/acsbiomaterials.3c00408

**Published:** 2023-04-19

**Authors:** Brittany N. Allen, Rion J. Wendland, Jacob D. Thompson, Budd A. Tucker, Kristan S. Worthington

**Affiliations:** † Roy J. Carver Department of Biomedical Engineering, College of Engineering, The University of Iowa, Iowa City, Iowa 52242-1002, United States; ‡ Department of Ophthalmology and Visual Sciences, Roy J. Carver College of Medicine, Institute for Vision Research, The University of Iowa, Iowa City, Iowa 52242-1002, United States

**Keywords:** photopolymerization, cell scaffold, microstructure, degradable polymer

## Abstract

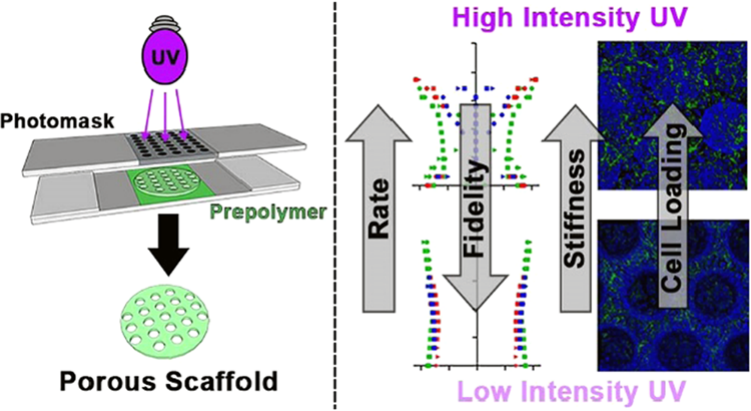

Engineered scaffolds are commonly used to assist in cellular transplantations, providing crucial support and specific architecture for a variety of tissue engineering applications. Photopolymerization as a fabrication technique for cell scaffolds enables precise spatial and temporal control of properties and structure. One simple technique to achieve a two-dimensional structure is the use of a patterned photomask, which results in regionally selective photo-cross-linking. However, the relationships between photopolymerization parameters like light intensity and exposure time and outcomes like structural fidelity and mechanical properties are not well-established. In this work, we used photopolymerization to generate degradable polycaprolactone triacrylate (PCLTA) scaffolds with a defined microstructure. We examined the impact of light intensity and exposure time on scaffold properties such as shear modulus and micropore structure. To assess feasibility in a specific application and determine the relationship between parameter-driven properties and cell loading, we cultured retinal progenitor cells on the PCLTA scaffolds. We found that light intensity and polymerization time directly impact the scaffold stiffness and micropore structure, which in turn influenced the cell loading capacity of the scaffold. Because material stiffness and topography are known to impact cell viability and fate, understanding the effect of scaffold fabrication parameters on mechanical and structural properties is critical to optimizing cell scaffolds for specific applications.

## Introduction

In tissue engineering and regenerative medicine, engineered scaffolds are commonly used to provide support and specific architecture to cells for transplantation or *in vitro* modeling. While there are many different factors that influence the effectiveness of cell scaffolds including material chemistry, mechanical properties, and structural design, the ability to precisely and reproducibly manufacture these scaffolds is an underlying challenge. As one example, transplantation of stem cell-derived retinal progenitor cells (RPCs) is an emerging therapeutic approach to mitigate irreversible blindness. However, the use of a scaffold that can protect cells from shear forces while also providing organizational cues is necessary for cell survival and function.^1−3^ Although advances in additive manufacturing, such as light-based three-dimensional (3D) printing, have enabled the creation of geometrically complex scaffolds for a range of biomedical applications, several limitations remain. For example, the precise manufacturing of small scaffold features (e.g., <20 μm) is achievable using advanced techniques like two-photon polymerization.^4,5^ However, in our experience, manufacturing a scaffold of relevant size for animal studies can take several days, which limits the plausibility of using this manufacturing technique for extensive preclinical studies.^4^ Faster manufacturing methods like stereolithography^6,7^ can overcome this limitation in some scenarios, but resolution is sacrificed, the scope of appropriate and available materials is narrow, and reliable instruments can be financially inaccessible.

Traditional photopolymerization is a fast and accessible method for fabricating thin polymer scaffolds and can be used with a broad range of chemistries, including many that are appropriate for biomedical applications.^8−10^ Spatial and temporal control is inherent to photopolymerization since reactions only occur when and where photons are actively supplied. Thus, patterns can easily be imposed upon the material by simply using a mask to block light from reaching the monomer or prepolymer solution. As with any fabrication method, it is critical to understand the impact of photopolymerization parameters on the scaffold’s final mechanical, structural, and biocompatibility properties. In this case, because photons diffract in the liquid solution, the fidelity of the final structure to the intended pattern depends on the space between photomask features as well as the photopolymerization reaction kinetics, as dictated by light intensity and exposure time.^11^ However, the relationships between fabrication parameters and scaffold properties, along with the cell response those properties induce, have not yet been quantified or described in the context of implantable, degradable biomaterials.

In this work, we use triacrylated poly(caprolactone) (PCLTA), a biocompatible synthetic polymer that is widely accepted as a suitable candidate material for cell scaffolds,^4,12−14^ to characterize how photopolymerization fabrication parameters, such as light intensity and exposure time, impact the stiffness, microporous structure, and biocompatibility properties of cell scaffolds. Understanding the interplay between fabrication parameters, scaffold characteristics, and resulting cellular responses provides additional tools for researchers, enabling the development of efficient and effective microstructured scaffolds for a wide range of biomedical applications, including retinal cell replacement therapy.

## Materials and Methods

### Prepolymer Preparation

Triacrylated poly(caprolactone) (PCLTA; Creative PEGWorks, Chapel Hill, NC) with a molecular weight of 300 g/mol was stored at −20 °C before use. Irgacure 2959 (Advanced BioMatrix, Carlsbad, CA) was used as the photoinitiator and was prepared in a stock solution at 100 mg/mL by solubilizing in ethanol. The photoinitiator stock solution was stored at 4 °C. The complete PCLTA solution was prepared by mixing 99 vol % PCLTA and 1 vol % of the stock Irgacure 2959 solution and stored at 4 °C until use within 2–3 days of mixing.

### Rheological Characterization

The shear stress response of the polymer scaffolds was assessed using a Malvern Kinexus Ultra+ Rheometer (Netzsch; Selb, Germany) equipped with an Omnicure S2000 UV light (320–500 nm filter, 8 mm adjustable collimating adapter) 3 cm away from the glass plate (Figure S1A). The PCLTA solution was warmed to room temperature and briefly vortexed to ensure homogeneity before loading 5 μL of PCLTA under an 8-mm-diameter upper plate geometry at a gap height of 0.05 mm. Oscillatory shear strain tests were conducted with a controlled strain of 0.1% and a frequency of 1 Hz. Shear modulus measurements were collected throughout UV photopolymerization at six different intensities (0.5, 1.0, 2.0, 3.0, 4.0, 5.0 W/cm^2^). For intensities of 1.0–5.0 W/cm^2^, irradiation on the rheometer occurred for a total time of 300 s. At an intensity of 0.5 W/cm^2^, irradiation time on the rheometer was increased to 360 s to facilitate full polymerization.

Final shear modulus values of each sample were calculated as previously described.^15^ Briefly, the data were fitted to the following equation^16^
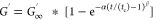
1where *G*′_∞_ is the infinite modulus parameter and α and β are curvature parameters.^16^*t*_c_ is the crossover time and was calculated using the point at which the shear storage modulus (*G*′) diverged from the shear loss modulus (*G*″), determined by an in-house MATLAB script. The modulus plateau point was recorded as the time (*t*_90%_) and modulus (*G*′_90%_) value where the shear storage modulus reached 90% of *G*′_∞_ (Figure S1B). Furthermore, as a proxy for the cross-linking rate, the slope of the shear storage modulus (d*G*′/d*t*) was calculated using the central difference method. As mechanical properties of a polymer are highly dependent on the extent of the reaction, this approach enabled us to visualize the cross-linking rate of the samples.

### Microstructured Scaffold Fabrication

Prior to scaffold fabrication, PCLTA solutions were warmed to room temperature and vortexed briefly to homogenize the solution. A mold was constructed using a standard glass slide and 50-μm-thick aluminum spacers. Spacers were placed on the two edges of the slide, and 40 μL of the prepolymer solution was pipetted in the center. A photomask (1.52-mm-thick sodalime glass, Applied Image Inc., Rochester, NY) with 75-μm-diameter chromium dots and 25 μm hexagonal spacing from the dot edge to edge was placed, chromium side down, over the glass slide, spacers, and solution. Binder clips were used to secure each end of the mold, which was placed 3 cm under the light source (OmniCure S2000 with a 320–500 nm filter and an 8 mm adjustable collimating adapter). PCLTA scaffolds were fabricated in triplicate at the six UV intensities tested (0.5, 1.0, 2.0, 3.0, 4.0, 5.0 W/cm^2^) for 350, 270, 200, 160, 140, and 125 s, respectively, based on minimum polymerization times determined from the photorheology results. Additionally, a group of samples were also irradiated at 0.5 W/cm^2^ for 300 s to examine scaffold properties below the calculated polymerization times.

### Removal of the Unpolymerized Material from Pores

After polymerization, scaffolds were washed in a series of dioxane (Macron Fine Chemicals) solutions to remove any hydrophobic, unpolymerized material. Iterative washes with 100/0, 75/25, 50/50, 25/75, and 0/100 of dioxane/PBS were performed for 1 h each.

### Pore Profile Analysis

After the unpolymerized material was removed by dioxane/PBS rinsing, PCLTA samples were imaged using a Nikon Eclipse Ti2 confocal microscope (Nikon Instruments Inc., Melville, NY). Z-stacks comprising 3.125 μm slices and spanning the entire scaffold thickness were collected using excitation at 488 nm, as PCLTA exhibits a high degree of autofluorescence at this wavelength. At each slice, the diameters of three randomly selected pores were measured using FIJI. Briefly, each z-stack file was converted to a series of 8-bit images, despeckled, and auto threshold was used to generate binary images. The slice number, major axis, minor axis, and circularity for each pore were obtained using the “analyze particles” function. Inclusion thresholds for area and circularity were set to 100–6000 μm^2^ and 0.25–1.0, respectively. To analyze the pore profiles, the average diameters of several pores in each scaffold were measured and plotted as a function of scaffold height. For scaffolds with closed pores, the average thickness of pore closure and maximum slope of the pore profile were quantified from these data. The number of closed slices was counted from confocal images of the scaffolds. The maximum slope of the pore shape was calculated by dividing the maximum change in pore diameter by the height of the scaffold over this region. In our convention, a slope of zero indicates that the pore profile is completely vertical (i.e., open), while a higher slope indicates that a pore that is more closed.

### Degradation

To form samples for the degradation study, a droplet of 60 μL of PCLTA with a photoinitiator (as described above) was polymerized between glass slides with two 1-mm-thick spacers using a light intensity of 2.5 W/cm^2^ for 240 s. The 1 mm thickness was chosen to ensure accurate measurements of the sample weight; 50-μm-thick samples were too small for their mass to be accurately and reproducibly measured. Samples were then placed in 1 mL of 1× PBS and incubated at 37 °C on a shaker plate at 50 rpm until their designated timepoint. At each timepoint, the wet mass of each sample was recorded, then each sample was placed in a preweighed microcentrifuge tube with 1 mL of deionized water, and frozen at −20 °C for at least 24 h. After freezing, samples were lyophilized using a Freezone 6 Liter Benchtop Freeze Dry System (Labconco, Kansas City, MO) at −60 °C and 0.31 mBarr for 24 h. The dry weight of each sample was recorded, and the relative dry mass and the sample swelling ratio were calculated using the equations below.
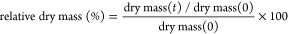
2
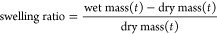
3

### Cell Culture

Immortalized rat retinal cells (R28, Applied Biological Materials, Richmond, VA) were cultured according to the manufacturer’s protocol. Briefly, cell culture flasks were coated with Matrigel Matrix (Corning, Bedford MA) for at least 30 min before cells were plated. DMEM/F12 was supplemented with 1% N2, 1% GlutaMAX, 10% FBS, and 1% Penicillin/Streptomycin by volume (Gibco, Thermo Fisher). Media were changed every 2–3 days. Once cells reached 80% confluency, they were delaminated with prewarmed trypsin containing 0.25% EDTA. After approximately 3 min, the delamination reaction was quenched by adding 2 times the volume of media to the volume of trypsin. Cells were centrifuged at 200 rcf for 3 min, and the supernatant was removed. The cell pellet was resuspended via gentle pipetting, and then the cell number was determined using a Countess II FL (Invitrogen, Thermo Fisher). Cells were plated at 40,000 cells/cm^2^ and incubated at 37 °C and 5% CO_2_.

### Scaffold Preparation for Cell Culture

After the unpolymerized material was removed by dioxane/PBS rinsing, 8-mm-diameter samples were obtained using a biopsy punch to ensure consistent scaffold size. Scaffolds were placed in 35% ethanol for one hour and then 70% ethanol for an additional hour to disinfect, followed by two 30 min sterile 1× PBS washes. Scaffolds were then soaked in complete media overnight. The following day, the media were removed, and scaffolds were secured to the bottom of each well of a 12-well plate using a stainless-steel washer and vacuum grease (Figure S2A). Next, 20 μL of Matrigel medium was added to each scaffold and incubated overnight.

### Cell Loading Assay

After scaffolds were prepared for cell culture, 200,000 cells in 20 μL of media were seeded on each scaffold. After seeding, scaffolds were incubated for 1 h to facilitate cell attachment. Then, 1 mL of media was added to each well and incubated for 48 h. Cell nuclei were stained using a NucRed Live 647 ReadyProbes reagent (Life Technologies, Thermo Fisher) at a concentration of two drops per mL of media and incubated for 15 min. Following incubation, media were removed from each well and 1 mL of 4% PFA diluted in 1× PBS was added. Samples were incubated in 4% PFA for 30 min at 4 °C, followed by three 10 min washes with washing buffer (1× PBS and 0.1% by volume Triton X). Alexa Fluor 488 phalloidin (Invitrogen) was added at 1:500 dilution in immunoblocking buffer, which consisted of 1× PBS with 5% donkey serum, 0.5% Triton X, 30 g/L bovine serum albumin, and 1.5 μL/mL 20 wt % sodium azide. Samples were incubated with phalloidin overnight at 4 °C. The following day, five additional 10 min washes were performed with washing buffer. Finally, samples were mounted with Aqua-Mount (Thermo Fisher), a coverslip was placed over the top of the sample, and samples were imaged using a Leica TCS SPE DMi8 inverted confocal microscope (Leica Microsystems, Wetzlar, Germany). The autofluorescence of the scaffold created interference during cell counting with the fluorescence signal alone, so single channel image stacks were depth-coded in order to improve counting accuracy (Figure S2B).

### Statistical Analysis

Scaffold storage moduli and polymerization times were extracted from UV photopolymerization rheology data based on the methodology described above and had four replicates for each group (*n* = 4). Crossover time and polymerization time with respect to light intensity were fit using an inverse square root regression, and the polymerized modulus values with respect to light intensity were fit using a power law regression, all using the sum of squares method. For pore profile analysis, the diameters of three pores on three independent scaffolds were measured from confocal z-stack images (*n* = 9). For cell loading, each group was performed in triplicate (*n* = 3). One-way ANOVA with Tukey’s multiple comparisons, each at a confidence interval of 95%, were used to determine differences in storage moduli, pore profile slopes, and cell number between intensity groups. Unpaired two-tailed *t*-tests at a confidence interval of 95% were used to compare the storage moduli and pore profile slopes between exposure time groups. Graphpad Prism software was used for all statistical analyses.

## Results and Discussion

**Figure 1:**
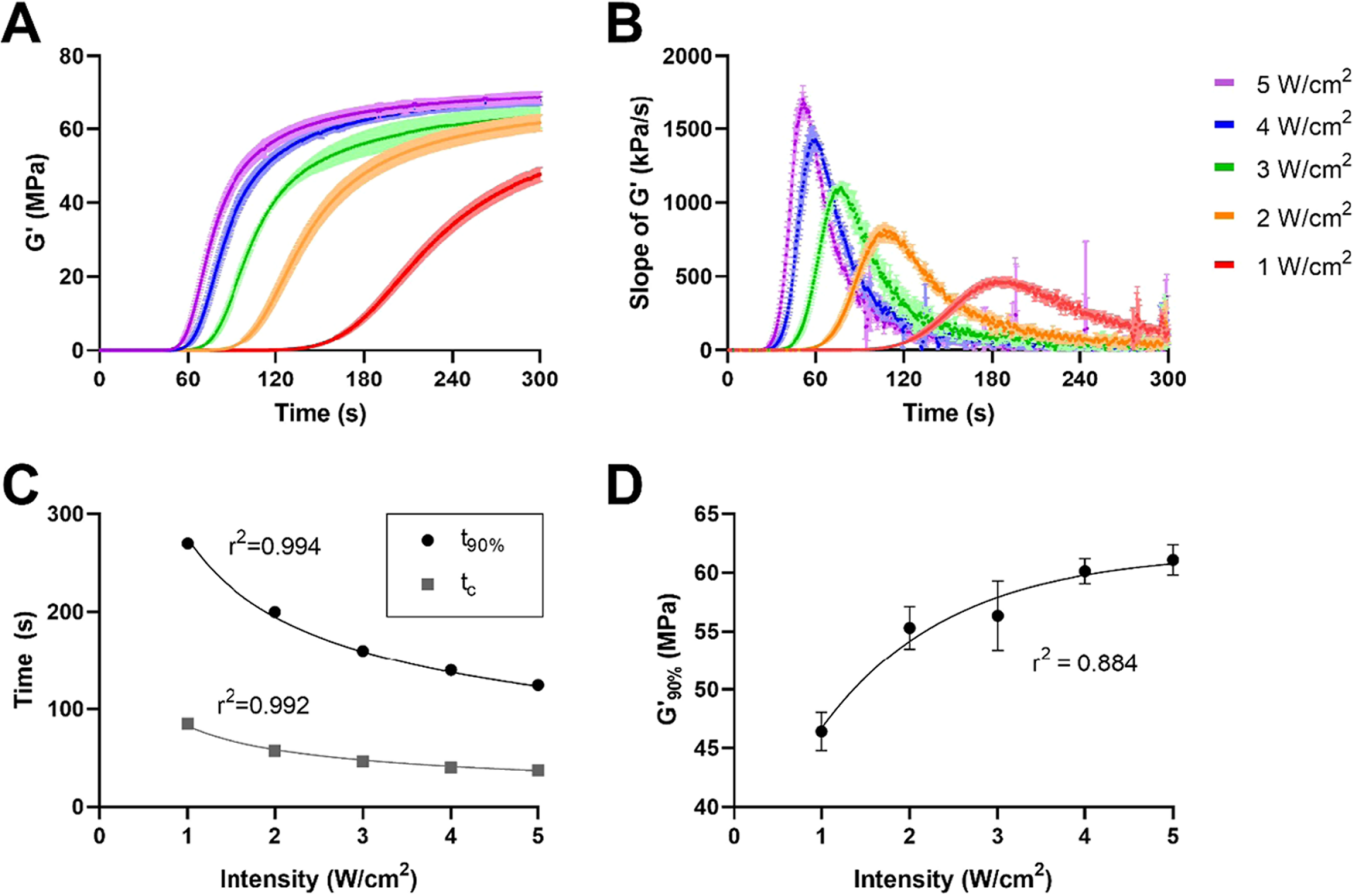
Polymerization rate, polymerization time, and storage modulus are affected by UV light intensity. (A) Shear modulus evolution during photopolymerization for each UV intensity. (B) Slope of the storage modulus over time for each UV intensity, which is used as a proxy measure of the polymerization rate. (C, D) Properties of PCLTA polymerized at an array of UV light intensities: polymerization (*t*_90%_) and crossover (*t*_c_) times with inverse square root lines of best fit (C) and polymerized modulus (*G*′_90%_), with a power law line of best fit (D). *n* = 4 for all groups; data points and error bars represent the mean and standard deviation, respectively.

Real-time photorheology was used to quantify PCLTA photopolymerization behavior and mechanical properties (Figure 1A). For all groups, photorheology data closely followed the empirical relationship given in eq 1, as indicated by all *R*^2^ values being greater than 0.98. As such, curve fit parameters *G*′_∞_, α, β, and *t*_c_, were extracted for each group (Table S1). Changes in the shear modulus (*G*′) during polymerization were used as a proxy to characterize the polymerization rate and cross-linking density. As expected, increasing light intensity caused increases in maximum polymerization rate (Figure 1B), following a linear relationship (Figure S3, Table S2). Cross-linking rate profiles also indicated that cross-linking began and reached a maximum rate earlier, with increasing light intensity (Figure 1C). As a result of these changes in kinetics, the polymerization time to reach the near-maximum modulus (*t*_90%_) was shorter as light intensity increased (∼350 vs ∼150 s for 0.5 vs 5 W/cm^2^; Figure 1C). Mathematically, these results closely follow traditional photopolymerization kinetics, in which the light intensity is inversely proportional to the polymerization rate by a square root factor.^17^ Indeed, the crossover (*t*_c_) and polymerization times (*t*_90%_) of the samples strongly follow this relationship with respect to the light intensity (Figure 1C and Table S3). In addition to the polymerization rate and time, increasing UV light intensity also had a significant effect on ultimate shear modulus values, which ranged from 38 to 61 MPa (Figure 1D). Unlike the polymerization time, the ultimate modulus values were not proportional to the light intensity by a square root factor (*R*^2^ = −0.65; analysis details available upon request). Rather, the modulus as a function of light intensity followed a logarithmic growth profile (*R*^2^ = 0.884, Figure 1D and Table S4). Specifically, the modulus increased with increasing light intensity, following a power law relationship (like *t*_c_ and *t*_90%_). Overall, these results further validate the important connection between polymerization kinetics and final polymer properties. Within a certain range (i.e., at submaximum conversion), modulation of UV light intensity can be used as a tool to control PCLTA scaffold stiffness, which in turn may be useful for instigating varied cell responses to the scaffold or achieving specific surgical handling properties.^15,18^

**Figure 2:**
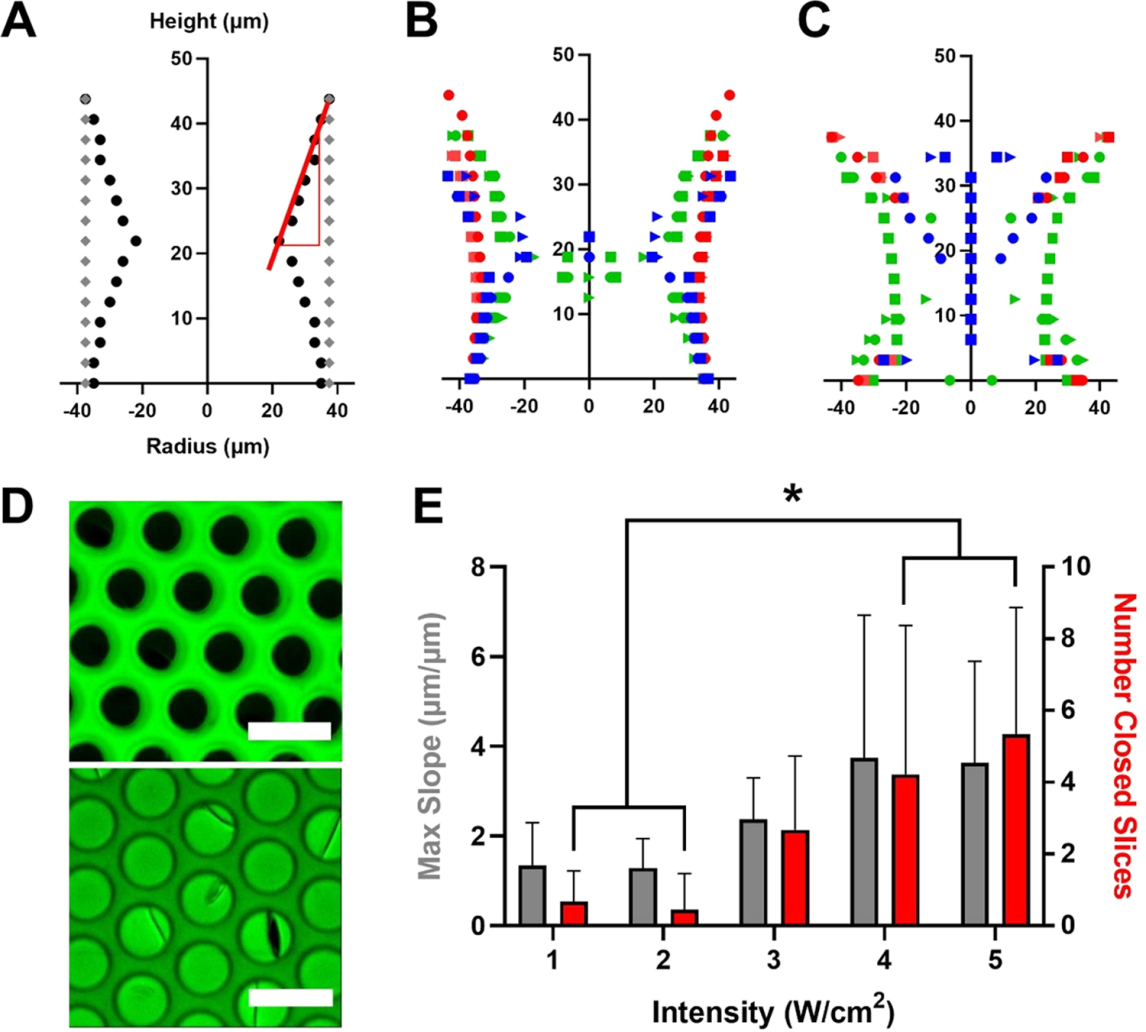
UV Light intensity impacts the micropore structure of PCLTA scaffolds. (A) Example schematic of pore profile reconstruction showing expected pore structure (gray), partially closed or “hourglass” pore structure (black), and slope of pore closure (red). (B) Reconstructed pore profiles of scaffolds polymerized at 1 W/cm^2^ show partially closed pores. (C) Reconstructed pore profiles of scaffolds polymerized at 5 W/cm^2^ show almost complete pore closure. Each color represents a unique scaffold, within which the varying shapes represent unique pores on the same scaffold (*n* = 9 pores total). (D) Representative images (collapsed confocal z-stacks) of scaffolds polymerized at 1 W/cm^2^ (top) and 5 W/cm^2^ (bottom). The scale bars represent 100 μm. (E) Maximum slope of pore profiles and closures for each light intensity.

To study the impact of UV light intensity on the fidelity of photo-micropatterned PCLTA, porous scaffolds were generated at various UV light intensities. Pore profiles were initially analyzed after washing scaffolds with 1× PBS (Figure S4). However, because PCLTA is not water soluble, aqueous washes were not sufficient to remove unpolymerized material from the scaffold pores. Thus, dioxane washes were implemented; based on our observations, the solvent was able to effectively remove unpolymerized PCLTA. Furthermore, these dioxane washes help to remove photoinitiator remnants from the polymerized scaffolds. In our past work, rigorous and comprehensive ISO10993 testing on the PCLTA scaffolds washed in this way showed no scaffold-induced toxicity or sensitization. Furthermore, exhaustive extraction studies demonstrated that residual monomer and photoinitiator levels were well below their corresponding margin of safety values.^4^ Overall, as UV light intensity increased, scaffolds contained an increasing number of partially or fully closed pores, and the vertical thickness of the pore closure increased (Figure 2A–E). At higher light intensities (3–5 W/cm^2^), most pores were completely closed midway through the scaffold, contributing to structures that resembled an hourglass shape. Statistical analysis showed that the number of closed slices (depth of pore closure) was significantly increased in the 4 and 5 W/cm^2^ groups compared to the 1 and 2 W/cm^2^ groups (Table S5). The quantified maximum slope of pore closure was also significantly impacted by light intensity (*p* = 0.0123, Table S5), with a general trend of increased slope with increased intensity. However, there were no significant differences detected in the post hoc analysis. Consistent with these findings, we and others have found that, although the use of high light intensities can enable enhanced *z*-precision, it can also cause aberrant cross-linking to occur in intended dark regions, negatively impacting resolution in the *xy*-plane.^4,5,19^ This is most likely due to light scattering that occurs through the liquid prepolymer solution. At low light intensities, only a few aberrant photons enter the “dark region” and thus negligible cross-linking in the intended pore regions. However, scattering increases with increasing light intensity; thus, the probability of photons entering the shadowed regions to initiate cross-linking also increases. Overall, these results indicate that light intensity significantly impacts the pore profile and can be used to modulate scaffold architecture.

**Figure 3:**
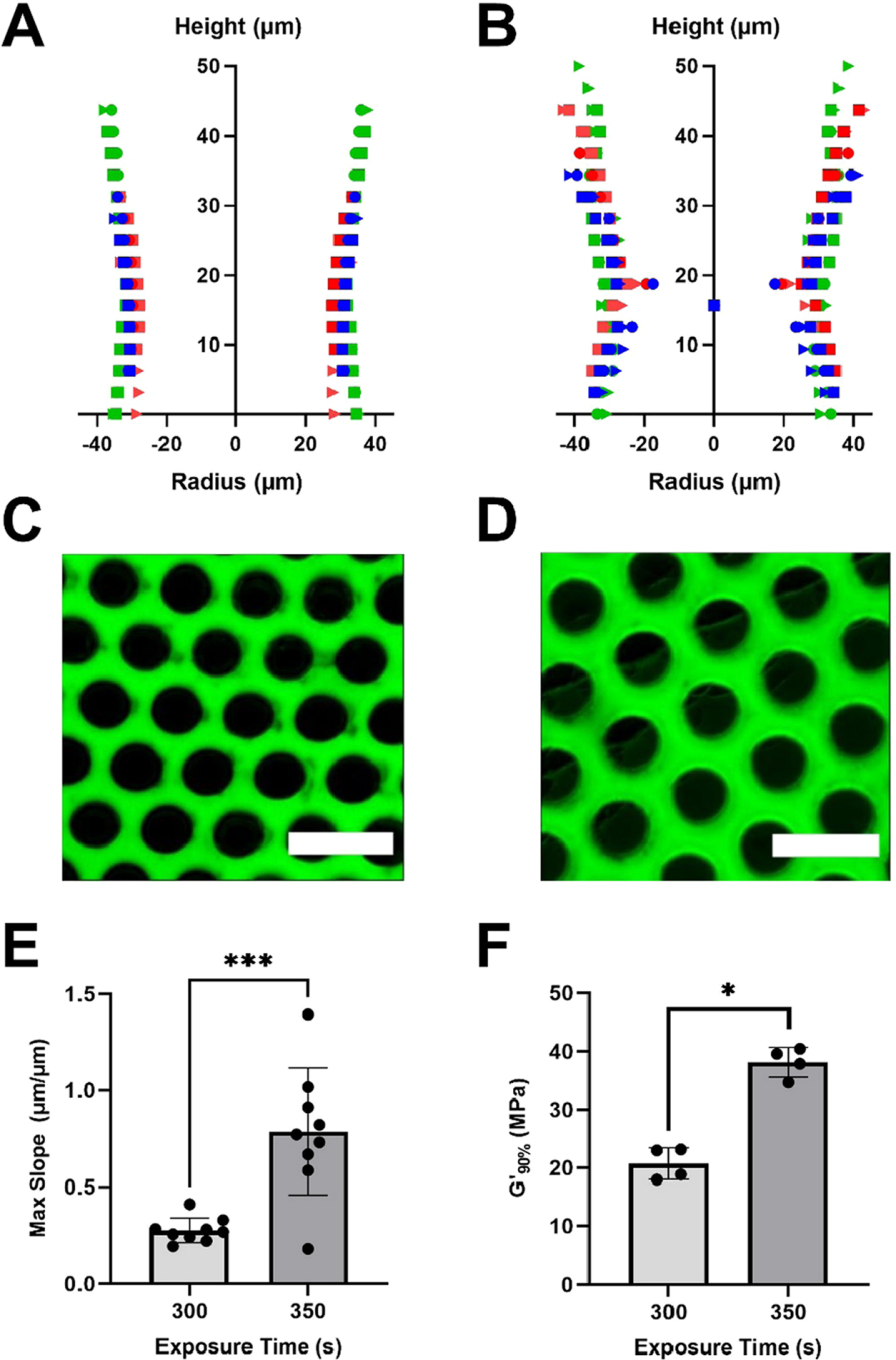
UV light exposure time impacts the scaffold micropore structure and modulus. Reconstructed pore profiles of PCLTA scaffolds polymerized with a UV light intensity of 0.5 W/cm^2^ for 300 (A) or 350 (B) seconds (*n* = 9 pores total for each group). Representative image (collapsed confocal z-stack) of the scaffold polymerized at 0.5 W/cm^2^ for 300 (C) or 350 (D) seconds. The scale bar represents 100 μm. (E) Maximum slope of pore closures for each exposure time (*n* = 9; data points and error bars represent the mean and standard deviation, respectively). (F) Polymerized storage modulus at each exposure time (*n* = 4; data points and error bars represent the mean and standard deviation, respectively). *p ≤ 0.05.

To fabricate scaffolds with pores that follow the photomask design, we tested two additional groups at a UV light intensity of 0.5 W/cm^2^. One group was polymerized for the full exposure time, 350 s, calculated as described above. The other group was polymerized for only 300 s, to reduce overpolymerization and thus decrease pore closure. Comparison of the pore profiles of these groups demonstrates that decreasing exposure time resulted in a significant decrease in the slope of the pore profile (*p* = 0.0003) and, by extension, increased fidelity to the intended scaffold structure. (Figure 3A–E). Additionally, decreasing the exposure time significantly decreased the scaffold modulus (*p* < 0.0001) from approximately 40 MPa to 20 MPa (Figure 3F). Shortening the exposure time of polymerization truncates the reaction, meaning that there is less time for cross-links to form. Based on photopolymerization reaction mechanisms and kinetics, we anticipate that this trend would hold true across all light intensities: at a given intensity, decreasing the exposure time is expected to decrease the scaffold stiffness while also maintaining architectural fidelity. However, accurately tuning properties by modulating exposure time may prove challenging at higher intensities, as the reaction proceeds much more rapidly. Regardless, modulating properties based on fabrication parameters could be an important tool for scaffold design and manufacturing, as material stiffness can play a role in cell fate decisions, particularly stem and progenitor cells.^20,21^ By only modulating the photopolymerization parameters (intensity or exposure time), scaffold mechanical properties and architecture can be altered without drastically changing the chemical composition or fabrication technique of the scaffold. Understanding the relationships between photopolymerization kinetics and final properties like architectural fidelity and stiffness is also critical for successful light-based 3D printing, especially in scenarios in which investigators wish to develop their own formulations without tedious iteration to determine optimal printing parameters.

**Figure 4:**
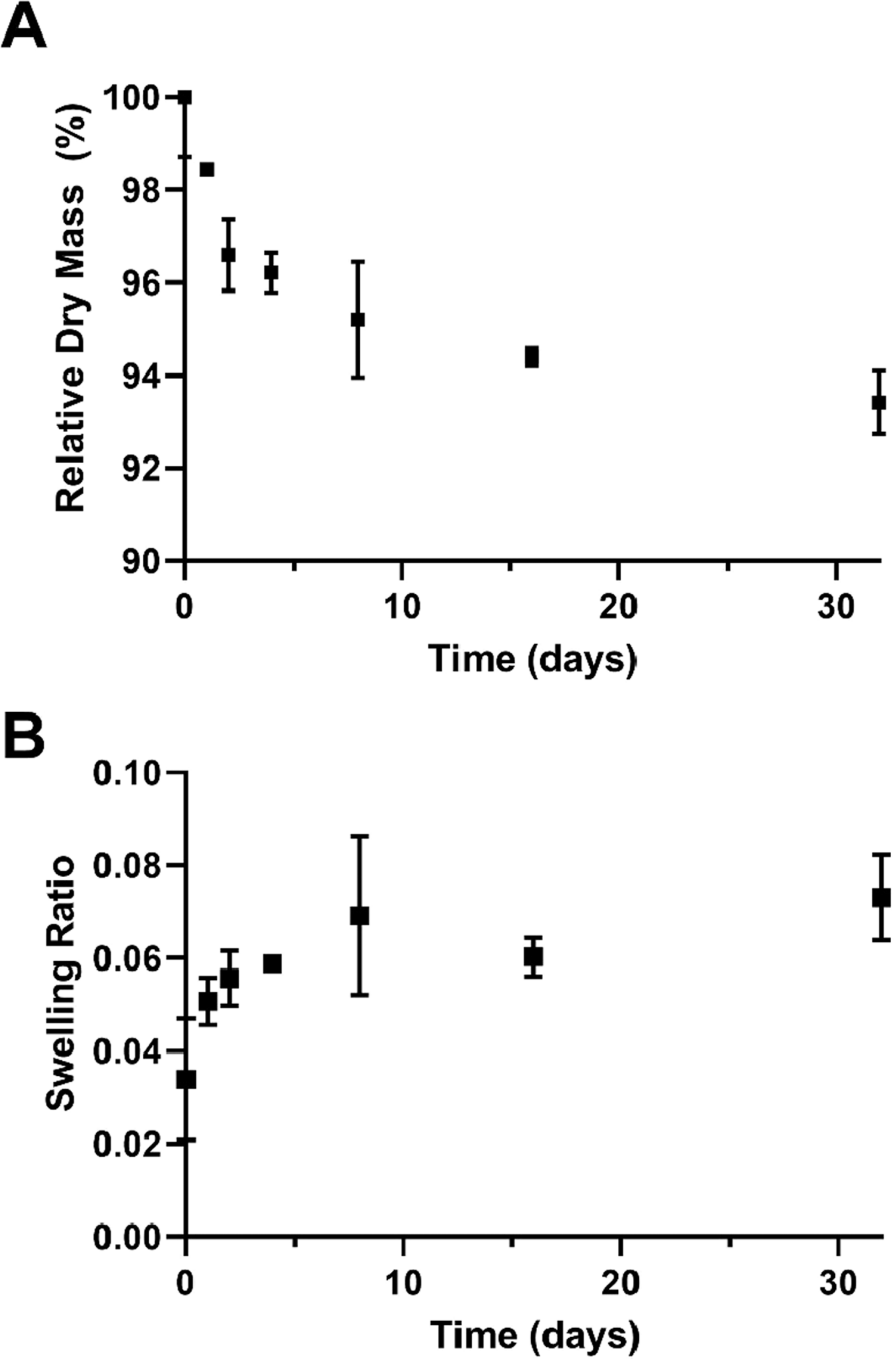
Photo-cross-linked PCLTA degrades slowly and experiences minimal swelling over 30 days. (A) Change in relative dry mass of PCLTA in PBS over 30 days. (B) Swelling of PCLTA over 30 days. *N* = 3 for all samples. Data points represent the mean, and error bars represent the standard deviation.

PCL is particularly attractive for tissue engineering and regenerative medicine applications due to its low toxicity and biodegradable nature. However, matching material degradation rates to new tissue growth or the desired therapeutic release rate is an important hurdle for any given application. Although a target degradation rate for a retinal cell delivery scaffold has yet to be established, we performed a simple degradation study here to serve as a baseline for further modulation in future work. Under the conditions in our study, PCLTA degraded slowly and steadily; only 6% of the dry mass was lost over the course of 30 days (Figure 4A). Swelling is also a critical factor to consider when developing cell delivery scaffolds, especially in the space-constricted environment of the retina. In our study, the swelling ratio was less than 0.1 over 32 days, which is negligible, especially in comparison to many other biomaterials (Figure 4B). It should be noted that scaffolds fabricated at different intensities and exposure times have different cross-linking densities, as evident from photorheology data (i.e., the final modulus increases with increasing intensity, see Figure 1). It is expected that the degradation rate of PCLTA scaffolds would be affected by the difference in cross-linking densities, and our prior work confirms this; in general, as the cross-linking density increases, the degradation rate decreases.^22^ However, we expect that these differences would not be significant for the samples in our study, specifically in the context of retinal transplantation procedures. Previous *in vivo* animal experiments have shown that PCL scaffolds remain intact following transplantation for up to 40 days in pigs and up to 6 months in rats.^4,23^ As PCL has a slow degradation rate relative to many other polymers such as poly(lactic acid) and poly(glycolic acid),^24,25^ differences in the cross-linking density based on light intensity and exposure time would not be expected to significantly affect the end application of a cell scaffold. In the future, longer studies would be necessary to determine the time PCLTA scaffolds take to completely degrade *in vivo* as cells’ hydrolytic processes may slightly accelerate degradation.

**Figure 5:**
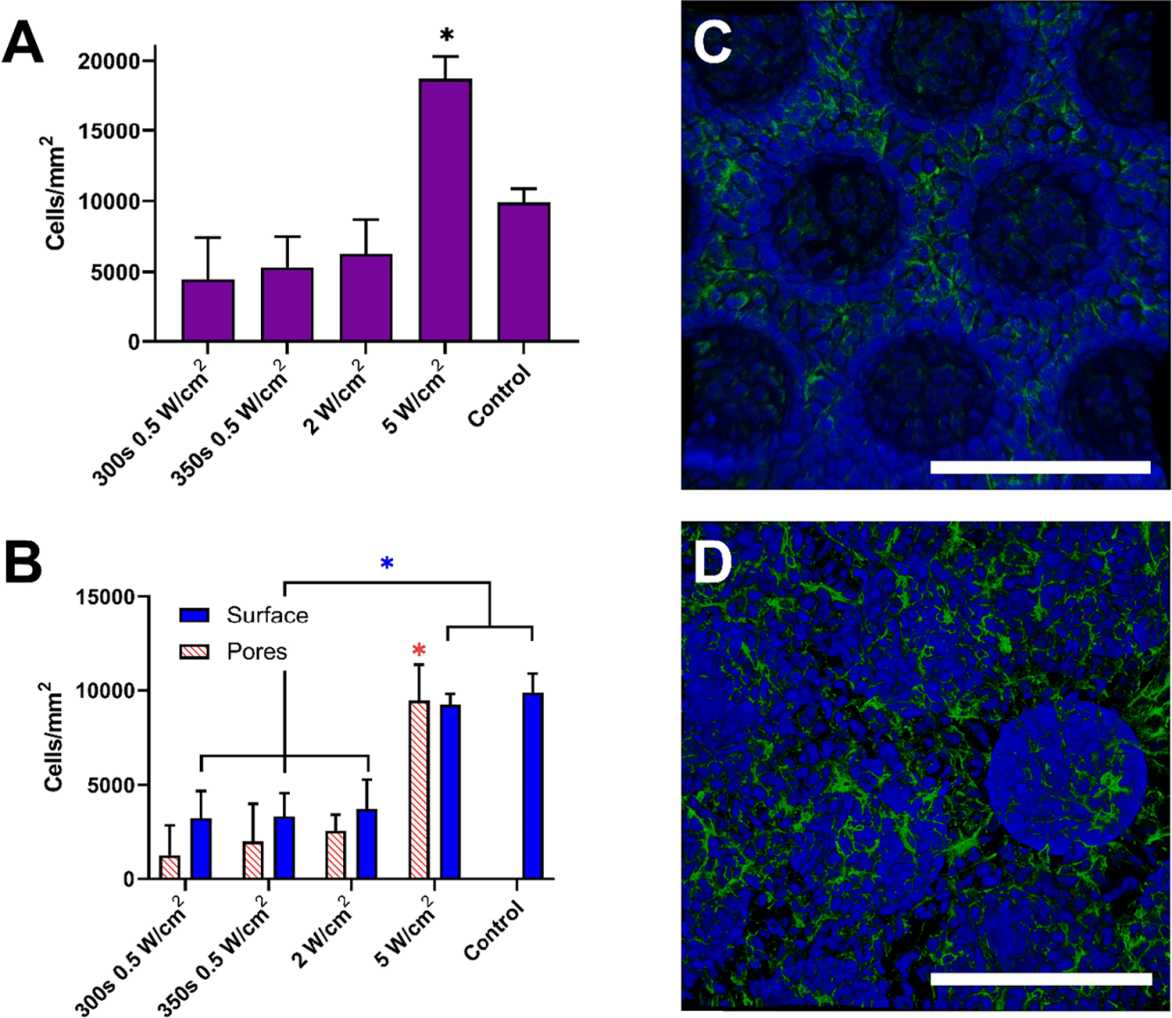
Micropore structure impacts the cell loading potential. (A) Total density of immortalized rat retinal progenitor cells (RPCs) on each scaffold. (B) Cell density on the surface of scaffold versus in pores. For (A) and (B), control samples were rat retinal progenitor cells seeded on a Matrigel-coated glass coverslips (*n* = 3, error bars represent the standard deviation, *p ≤ 0.05). (C) Representative image of rat RPCs on a scaffold polymerized for 300 s at 0.5 W/cm^2^. (D) Representative image of the PCLTA scaffold polymerized for 125 s (*t*_90%_) at 5 W/cm^2^. Blue represents cell nuclei (DAPI), and green represents F-actin (Phalloidin). All scale bars represent 100 μm.

The ability to control the micropore structure and the consistency of the micropore structure are likely to be important for controlling cell loading density. In this study, the impacts of the micropore structure on cell loading were examined by seeding immortalized retinal progenitor cells onto scaffolds polymerized at different UV light intensities. Due to the autofluorescence of PCLTA, cells were counted using depth-coded images, which enabled the quantification of cells in pores as opposed to those on the surface (Figure S2). Scaffolds with closed pores (5 W/cm^2^) retained significantly more cells than scaffolds with open pores (0.5 and 2 W/cm^2^) and the control, which was a glass coverslip coated with Matrigel (Figure 5A, Table S6). As expected, scaffolds polymerized at 5 W/cm^2^ also carried a significantly higher density of cells within the pores than scaffolds polymerized at lower light intensities (Figure 5B, Table S6). This observation is likely due to cells’ ability to settle and aggregate in the partially closed pores, which seem to act as microwells in this scenario. In contrast, in sample groups with open pores, cells seemed to attach to or migrate along the inner surface of the pores, leading to an apparent ring of cells when viewing collapsed z-stack images (Figure 5C). In addition to carrying a greater number of cells within the pores, scaffolds polymerized at 5 W/cm^2^ (Figure 5D) also had a significantly higher density of cells on their surface compared to other scaffold groups but not significantly different than the control (Figure 5B, Table S6). We hypothesize that this difference is due to more efficient loading; it can be assumed that fewer cells were lost during the loading process for scaffolds with closed or partially closed pores compared to scaffolds with mostly open pores. Although we considered the possibility that the scaffold modulus could have played a role in observed differences in cell density on the scaffold surface, this hypothesis is not supported by the data since the open-pore scaffolds in our study (two at 0.5 W/cm^2^ and one at 2 W/cm^2^) each had a distinct modulus (see Figure 1) but were not statistically different with respect to cell loading (Figure 5B).

Due to the autofluorescent and opaque nature of cross-linked PCLTA, characterizing cell morphology as a function of the scaffold microstructure or modulus was not plausible in this study. Regardless, the cell line we selected here is immortalized and thus has limited capacity for further differentiation toward mature retinal phenotypes. Studies using human pluripotent stem cell-derived retinal progenitor cells to elucidate the intricate relationships between the scaffold modulus, degradation time, and cell fate and function would further advance the possibility of employing microstructured PCLTA scaffolds for retinal cell delivery. However, the results we present here highlight the possibility of using photopolymerization parameters of microporous scaffolds to meet the needs of a specific application. For example, we found that the hourglass shape of pores on 5 W/cm^2^ scaffolds is more conducive to cell retention than cylindrical pores, likely because cells are less likely to fall through the pores and settle on the tissue culture plastic below the scaffold. Furthermore, the layer of PCLTA remaining in the pores is thin and likely less densely cross-linked than the surrounding material due to having received indirect (and therefore less) light exposure. Thus, while this thin membrane of PCLTA could be used to promote *in vitro* cell loading, it is also expected to degrade more quickly *in vivo* than the rest of the scaffold due to its differentially lower cross-linking density. This selective degradation could be leveraged to promote organized cell integration with the native tissue after cells are safely delivered to the target tissue.

The work we present here is an integral start to understanding the ways in which fabrication parameters can influence photopolymerized scaffold mechanical properties and architecture. There are several additional properties that must be thoroughly investigated to further refine scaffold design for specific applications such as retinal cell replacement therapy. For example, coating chemistry, degradation rate, and cell seeding density are each vital to the success of tissue engineering scaffolds. Furthermore, the viability and gene expression of cells plated on photopolymerized PCLTA scaffolds with a variety of micropore structures will also be important parameters for finalizing scaffold design and determining the optimal seeding density for future clinical studies.

## Conclusions

This study supports our hypothesis that adjustments in photopolymerization parameters can be used as tools to modulate mechanical and microstructural properties of scaffolds and that tuning of these properties impacts cell loading capabilities of the scaffold. Although we tested this hypothesis in the context of retinal engineering, our methodology could be applied to other photopolymerized scaffolds for a wide variety of tissue engineering or cell delivery purposes. These concepts are also relevant for precisely predicting and controlling the properties of scaffolds fabricated using light-based 3D printing. Ultimately, understanding the connections between photopolymerization kinetics and properties of biomaterials advances the field by enabling scientists and engineers to develop and employ custom resins without having to exhaustively iterate to identify optimal fabrication parameters.

## Supporting Information Available

Additional methodology descriptions, detailed results from curve fitting, regressions, statistical analyses, pore profile data, and representative fluorescent images of scaffolds with and without dioxane rinsing (PDF)

## References

[ref1] Burnight E. R.; Giacalone J. C.; Cooke J. A.; Thompson J. R.; Bohrer L. R.; Chirco K. R.; Drack A. V.; Fingert J. H.; Worthington K. S.; Wiley L. A.; Mullins R. F.; Stone E. M.; Tucker B. A. CRISPR-Cas9 genome engineering: Treating inherited retinal degeneration. Prog. Retinal Eye Res. 2018, 65, 28–49. 10.1016/j.preteyeres.2018.03.003. PMC8210531 29578069

[ref2] Tomita M.; Lavik E.; Klassen H.; Zahir T.; Langer R.; Young M. J. Biodegradable polymer composite grafts promote the survival and differentiation of retinal progenitor cells. Stem Cells 2005, 23, 1579–1588. 10.1634/stemcells.2005-0111. 16293582

[ref3] Lavik E. B.; Klassen H.; Warfvinge K.; Langer R.; Young M. J. Fabrication of degradable polymer scaffolds to direct the integration and differentiation of retinal progenitors. Biomaterials 2005, 26, 3187–3196. 10.1016/j.biomaterials.2004.08.022. 15603813

[ref4] Thompson J. R.; Worthington K. S.; Green B. J.; Mullin N. K.; Jiao C.; Kaalberg E. E.; Wiley L. A.; Han I. C.; Russell S. R.; Sohn E. H.; Guymon C. A.; Mullins R. F.; Stone E. M.; Tucker B. A. Two-photon polymerized poly(caprolactone) retinal cell delivery scaffolds and their systemic and retinal biocompatibility. Acta Biomater. 2019, 94, 204–218. 10.1016/j.actbio.2019.04.057. 31055121 PMC6659122

[ref5] Shrestha A.; Allen B. N.; Wiley L. A.; Tucker B. A.; Worthington K. S. Development of High-Resolution Three-Dimensional-Printed Extracellular Matrix Scaffolds and Their Compatibility with Pluripotent Stem Cells and Early Retinal Cells. J. Ocul. Pharmacol. Ther. 2020, 36, 42–55. 10.1089/jop.2018.0146. 31414943 PMC7476392

[ref6] Steedman M. R.; Tao S. L.; Klassen H.; Desai T. A. Enhanced differentiation of retinal progenitor cells using microfabricated topographical cues. Biomed. Microdevices 2010, 12, 363–369. 10.1007/s10544-009-9392-7. 20077017 PMC2859162

[ref7] McUsic A. C.; Lamba D. A.; Reh T. A. Guiding the morphogenesis of dissociated newborn mouse retinal cells and hES cell-derived retinal cells by soft lithography-patterned microchannel PLGA scaffolds. Biomaterials 2012, 33, 1396–1405. 10.1016/j.biomaterials.2011.10.083. 22115999 PMC3249403

[ref8] Choi J. R.; Yong K. W.; Choi J. Y.; Cowie A. C. Recent advances in photo-crosslinkable hydrogels for biomedical applications. Biotechniques 2019, 66, 40–53. 10.2144/btn-2018-0083. 30730212

[ref9] Nguyen Q. T.; Hwang Y.; Chen A. C.; Varghese S.; Sah R. L. Cartilage-like mechanical properties of poly (ethylene glycol)-diacrylate hydrogels. Biomaterials 2012, 33, 6682–6690. 10.1016/j.biomaterials.2012.06.005. 22749448 PMC3572364

[ref10] Khoshakhlagh P.; Moore M. J. Photoreactive interpenetrating network of hyaluronic acid and Puramatrix as a selectively tunable scaffold for neurite growth. Acta Biomater. 2015, 16, 23–34. 10.1016/j.actbio.2015.01.014. 25617804

[ref11] Tuft B. W.; Li S. F.; Xu L. J.; Clarke J. C.; White S. P.; Guymon B. A.; Perez K. X.; Hansen M. R.; Guymon C. A. Photopolymerized microfeatures for directed spiral ganglion neurite and Schwann cell growth. Biomaterials 2013, 34, 42–54. 10.1016/j.biomaterials.2012.09.053. 23069708 PMC4306579

[ref12] Yao J.; Ko C. W.; Baranov P. Y.; Regatieri C. V.; Redenti S.; Tucker B. A.; Mighty J.; Tao S. L.; Young M. J. Enhanced Differentiation and Delivery of Mouse Retinal Progenitor Cells Using a Micropatterned Biodegradable Thin-Film Polycaprolactone Scaffold. Tissue Eng., Part A 2015, 21, 1247–1260. 10.1089/ten.tea.2013.0720. 25517296 PMC4394889

[ref13] Christiansen A. T.; Tao S. L.; Smith M.; Wnek G. E.; Prause J. U.; Young M. J.; Klassen H.; Kaplan H. J.; la Cour M.; Kiilgaard J. F. Subretinal Implantation of Electrospun, Short Nanowire, and Smooth Poly(epsilon-caprolactone) Scaffolds to the Subretinal Space of Porcine Eyes. Stem Cells Int. 2012, 2012, 454295 10.1155/2012/454295. 22550509 PMC3328168

[ref14] Steedman M. R.; Tao S. L.; Klassen H.; Desai T. A. Enhanced differentiation of retinal progenitor cells using microfabricated topographical cues. Biomed Microdevices 2010, 12, 363–369. 10.1007/s10544-009-9392-7. 20077017 PMC2859162

[ref15] Wendland R. J.; Jiao C.; Russell S. R.; Han I. C.; Wiley L. A.; Tucker B. A.; Sohn E. H.; Worthington K. S. The effect of retinal scaffold modulus on performance during surgical handling. Exp. Eye Res. 2021, 207, 108566 10.1016/j.exer.2021.108566. 33838142 PMC8187337

[ref16] Cao X. J.; Cummins H. Z.; Morris J. F. Structural and rheological evolution of silica nanoparticle gels. Soft Matter 2010, 6, 5425–5433. 10.1039/c0sm00433b.

[ref17] Odian G. Principles of Polymerization, 4th ed.; John Wiley & Sons, Inc, 2004.

[ref18] Alvarez Y.; Smutny M. Emerging Role of Mechanical Forces in Cell Fate Acquisition. Front. Cell Dev. Biol. 2022, 10, 864522 10.3389/fcell.2022.864522. 35676934 PMC9168747

[ref19] Lim J.; Lee S.; Kim J. Structural dimensions depending on light intensity in a 3D printing method that utilizes in situ light as a guide. Micro and Nano Systems Lett. 2020, 8, 9 10.1186/s40486-020-00111-2.

[ref20] Murphy W. L.; McDevitt T. C.; Engler A. J. Materials as stem cell regulators. Nat. Mater. 2014, 13, 547–557. 10.1038/nmat3937. 24845994 PMC4163547

[ref21] Vining K. H.; Mooney D. J. Mechanical forces direct stem cell behaviour in development and regeneration. Nat. Rev. Mol. Cell Biol. 2017, 18, 728–742. 10.1038/nrm.2017.108. 29115301 PMC5803560

[ref22] Green B. J.; Worthington K. S.; Thompson J. R.; Bunn S. J.; Rethwisch M.; Kaalberg E. E.; Jiao C. H.; Wiley L. A.; Mullins R. F.; Stone E. M.; Sohn E. H.; Tucker B. A.; Guymon C. A. Effect of Molecular Weight and Functionality on Acrylated Poly(caprolactone) for Stereolithography and Biomedical Applications. Biomacromolecules 2018, 19, 3682–3692. 10.1021/acs.biomac.8b00784. 30044915

[ref23] Han I. C.; Bohrer L. R.; Gibson-Corley K. N.; Wiley L. A.; Shrestha A.; Harman B. E.; Jiao C. H.; Sohn E. H.; Wendland R.; Allen B. N.; Worthington K. S.; Mullins R. F.; Stone E. M.; Tucker B. A. Biocompatibility of Human Induced Pluripotent Stem Cell-Derived Retinal Progenitor Cell Grafts in Immunocompromised Rats. Cell Transplant. 2022, 31, 1–25. 10.1177/09636897221104451. PMC9247396 35758274

[ref24] Jeong S. I.; Kim B. S.; Kang S. W.; Kwon J. H.; Lee Y. M.; Kim S. H.; Kim Y. H. In vivo biocompatibilty and degradation behavior of elastic poly(L-lactide-co-epsilon-caprolactone) scaffolds. Biomaterials 2004, 25, 5939–5946. 10.1016/j.biomaterials.2004.01.057. 15183608

[ref25] Sun H. F.; Mei L.; Song C. X.; Cui X. M.; Wang P. Y. The in vivo degradation, absorption and excretion of PCL-based implant. Biomaterials 2006, 27, 1735–1740. 10.1016/j.biomaterials.2005.09.019. 16198413

