# Supplementary material for: Photopolymerization Parameters Influence Mechanical, Microstructural, and Cell Loading Properties of Rapidly Fabricated Cell Scaffolds
Source: ACS Biomater Sci Eng. 2023 Apr 19;9(5):2663–71. doi: 10.1021/acsbiomaterials.3c00408 (PMC10170473; doi:10.1021/acsbiomaterials.3c00408)
Supplement: Supplementary file 1 — ab3c00408_si_001.pdf [file ab3c00408_si_001.pdf]

SUPPORTING INFORMATION

Photopolymerization parameters influence mechanical, microstructural, and cell loading properties of rapidly fabricated cell scaffolds

Brittany N Allen[1#], Rion J Wendland[1,2#], Jacob D Thompson[1], Budd A Tucker[2], Kristan S Worthington[1,2]*

*Corresponding author. Email address: kristan-worthington@uiowa.edu

#Co-first authors

AFFILIATIONS

[1] Roy J. Carver Department of Biomedical Engineering, College of Engineering, The University of Iowa, Iowa City, IA, USA 52242-1002.

[2] Institute for Vision Research, Department of Ophthalmology and Visual Sciences, Roy J. Carver College of Medicine, The University of Iowa, Iowa City, IA, USA 52242-1002.

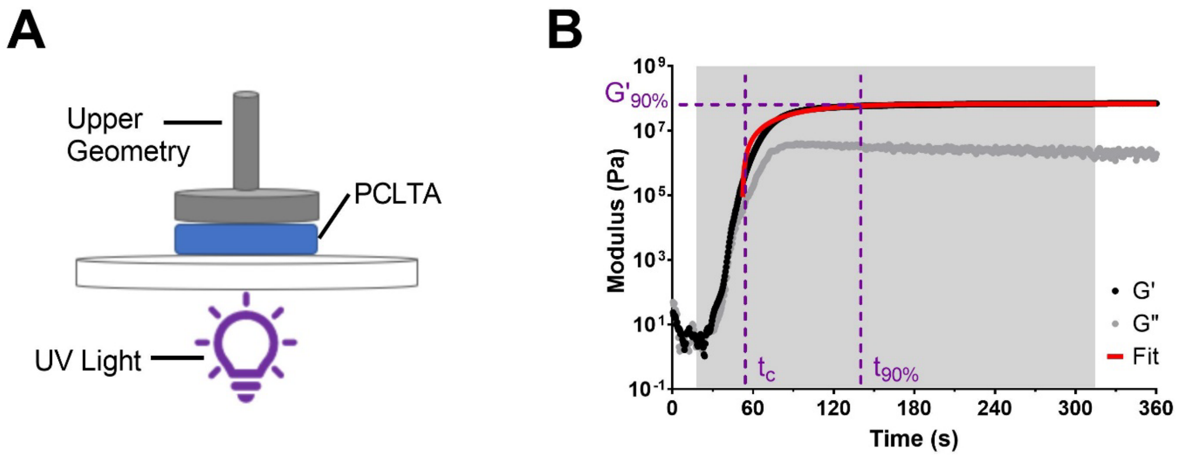

Figure S1: Photorheology can be used to measure polymerization rate and shear modulus throughout photopolymerization. **A**) Diagram of photo-rheometer setup. **B**) Example of polymerization curve generated by Matlab code using photorheology data. Shaded area indicates period in which the UV light was on during the data collection. G' (black) and G'' (grey) represent storage and loss modulus, respectively, while the red line shows the best fit of storage modulus. Dashed lines indicate the crossover time (t_c), polymerization time ($t_{90\%}$), and corresponding polymerized modulus ($G'_{90\%}$).

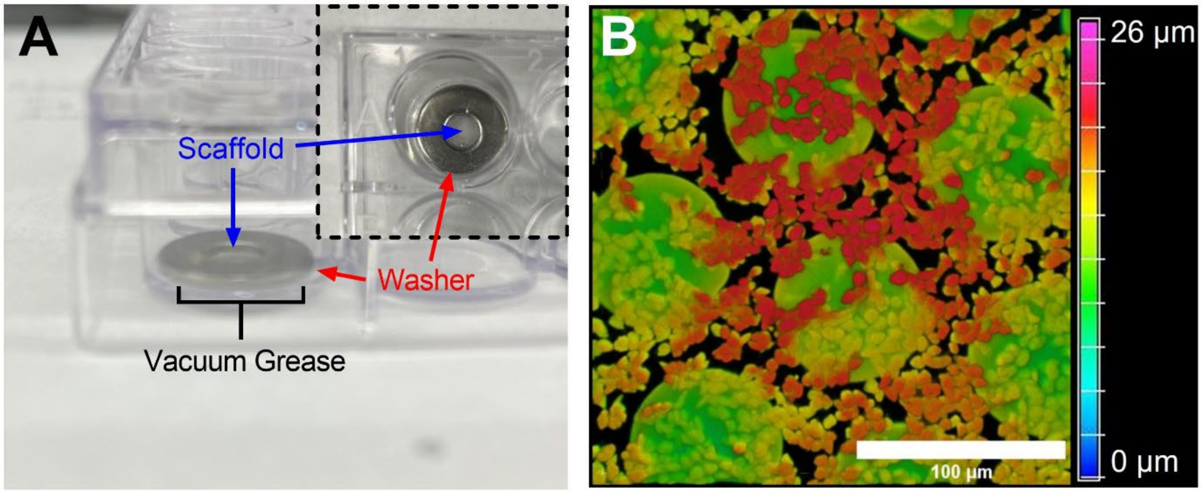

Figure S2: Methodology for cell culture experiments. **A**) A stainless-steel washer was used to ensure PCLTA scaffolds remained immobile on the bottom of the plate during cell culture. **B**). Representative depth-coded image used for counting of cells in the pores and on the surface of the scaffold.

Table S1. Curve fitting parameters (G_∞, α, β) for the polymerization fitting equation (**Equation 1**) as well as the crossover time (t_c) and the corresponding r^2 fit; corresponds with data in **Figure 1**. Each value represents mean ± standard deviation.

Intensity (W/cm^2)	G_∞ (MPa)	α	β	t_c (s)	r^2
**0.5**					
**t = 300s**	23.04 ± 2.61	0.40 ± 0.04	4.88 ± 0.15	136.25 ± 2.49	$0.9996 \pm 1.15*10^{-4}$
**t = 350s**	42.36 ± 2.46	0.24 ± 0.01	3.78 ± 0.09	132 ± 5.01	$0.9996 \pm 1.72*10^{-4}$
**1**	51.58 ± 1.56	0.24 ± 0.01	3.05 ± 0.04	91.75 ± 0.43	$0.9979 \pm 2.34*10^{-4}$
**2**	61.45 ± 1.75	0.41 ± 0.02	2.13 ± 0.03	63.5 ± 1.00	$0.9921 \pm 4.89*10^{-4}$
**3**	62.60 ± 2.85	0.66 ± 0.09	1.68 ± 0.13	54.5 ± 1.58	$0.9870 \pm 29.18*10^{-4}$
**4**	66.81 ± 1.03	0.94 ± 0.08	1.39 ± 0.06	50.63 ± 1.67	$0.9869 \pm 11.59*10^{-4}$
**5**	67.88 ± 1.24	0.95 ± 0.08	1.36 ± 0.09	45.63 ± 1.67	$0.9845 \pm 8.96*10^{-4}$

Table S2. Linear regression (y = m*x + b) of the relationship between maximum slope of storage modulus (i.e., crosslinking rate) and UV light intensity; corresponding to **Figure 1B** and **Figure S3**.

**Parameter**	**Best Fit**	**95% CI**
m	308.3	295.3 to 321.3
b	199.0	159.7 to 238.3
**Goodness of Fit**		
R squared	0.9910	
Sy.x	48.76	
**One-Way ANOVA – is Slope Significantly Non-Zero?**		
F	2432	
p-value	<0.0001	

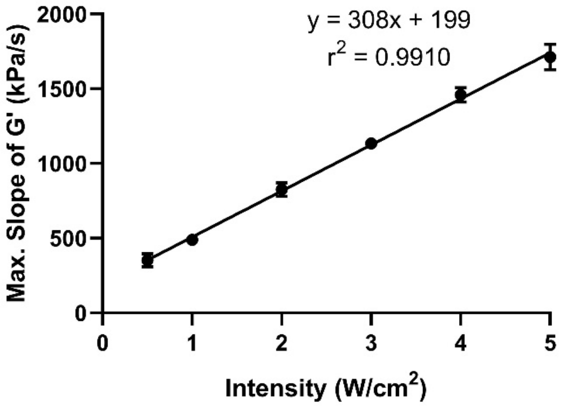

Figure S3. Linear relationship between maximum slope of storage modulus (i.e., crosslinking rate) and UV light intensity; corresponding to **Figure 1A** and **Table S2**.

Table S3. Non-linear regression ($y = a*x^{0.5}$) of the relationship between time (crossover time, t_c and time at full polymerization, $t_{90\%}$) and UV light intensity (corresponding to **Figure 1C**). This relationship between exposure time and intensity is derived from the photopolymerization rate equation.

**Crossover Time (t_c)**		
**Parameter**	**Best Fit**	**95% CI**
a	275.4	272.6 to 278.2
**Goodness of Fit**		
R squared	0.9941	
Sy.x	4.099	
**Time at Max. Rate (t_{max})**		
**Parameter**	**Best of Fit**	**95% CI**
a	82.67	81.57 to 83.77
**Goodness of Fit**		
R squared	0.9920	
Sy.x	1.588	

Table S4. Non-linear regression [exponential plateau, $y = y_m-(y_m-y_0)*e^{-kx}$] of the effect of light intensity on final storage modulus of photo crosslinked PCLTA (corresponding to **Figure 1D**).

**Parameter**	**Best Fit**	**95% CI**
y_m	61.73	59.14 to 68.94
y_0	31.87	9.644 to 41.64
k	0.6851	0.2819 to 1.187
**Goodness of Fit**		
R squared	0.8837	
Sy.x	2.014	

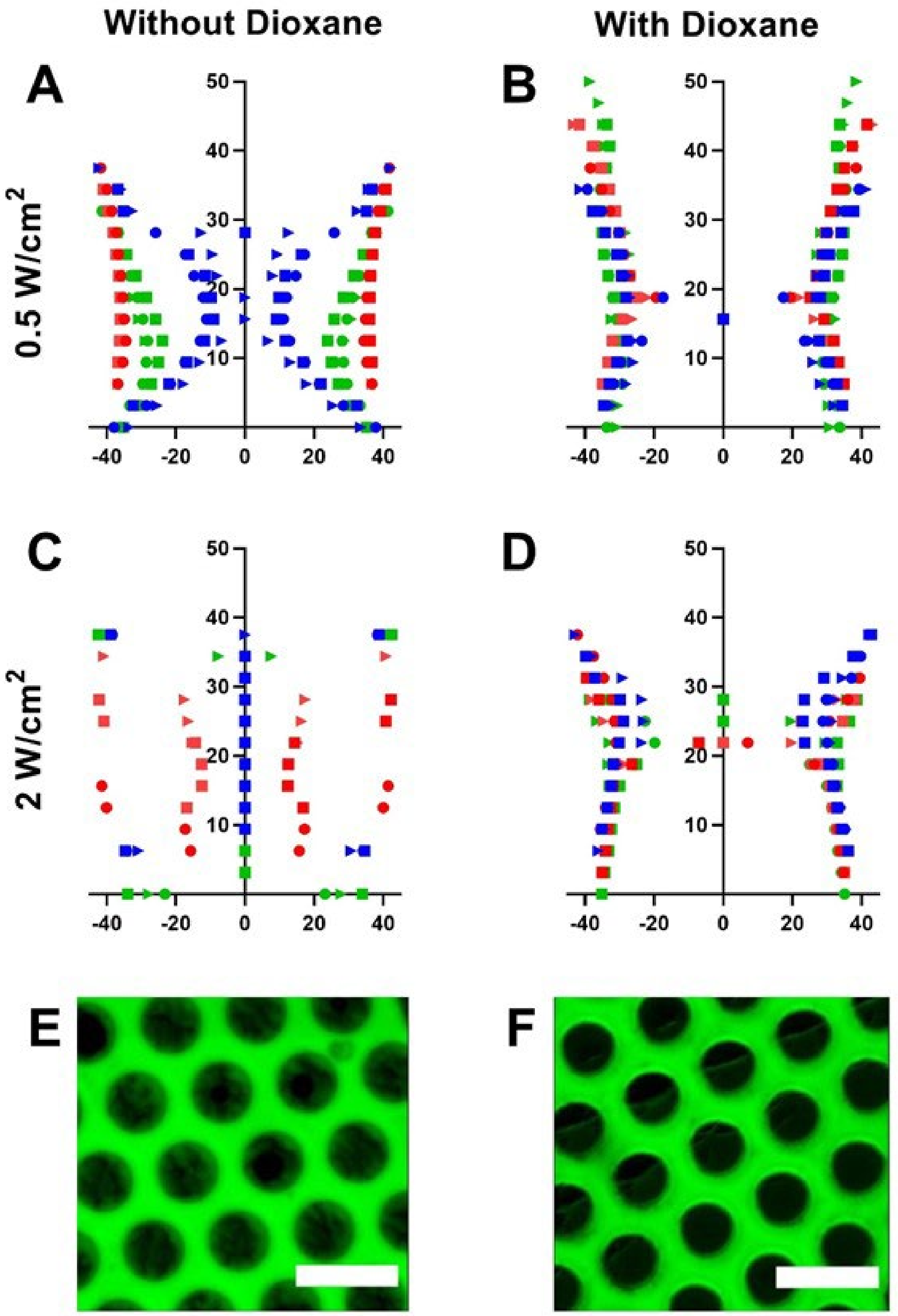

Figure S4: Washing scaffolds with dioxane removes unpolymerized PCL and opens pores. **A-D)** Reconstructions of pore profiles of scaffolds with and without dioxane washes (n=9). **E-F)** Representative images (collapsed confocal z-stacks) of scaffolds polymerized at 0.5 W/cm^2 with and without dioxane washes. Scale bars represent 100 µm. Note: Data with dioxane washes is also shown in manuscript.

Table S5: Results of statistical tests comparing pore profiles of scaffolds polymerized at an array of UV light intensities (results correspond to data in **Figure 2E**).

**MAXIMUM SLOPE**				
**One-way ANOVA:** p = 0.0123				
**Tukey's Multiple Comparisons Tests**				
Intensity (W/cm^2)	1.0	2.0	3.0	4.0
2.0	>0.9999	-	-	-
3.0	0.7704	0.7319	-	-
4.0	0.0671	0.0574	0.5289	-
5.0	0.0878	0.0755	0.6043	>0.9999
**THICKNESS OF CLOSURE**				
**One-way ANOVA:** p = 0.0009				
**Tukey's Multiple Comparisons Tests**				
Intensity (W/cm^2)	1.0	2.0	3.0	4.0
2.0	0.9998	-	-	-
3.0	0.5140	0.4084	-	-
4.0	0.0542	0.0355	0.7315	-
5.0	0.0055	0.0033	0.2333	0.9019

Table S6: Results of statistical tests comparing cell loading capabilities of scaffolds polymerized at an array of UV light intensities (results correspond to data in **Figure 5A-B**).

**TOTAL CELL DENSITY**				
**One-way ANOVA:** p < 0.0001				
**Tukey's Multiple Comparisons Tests**				
Intensity (W/cm^2)	Control	0.5 (300 s)	0.5 (350 s)	2.0
0.5 (300 s)	0.0652	-	-	-
0.5 (350 s)	0.1360	0.9873	-	-
2.0	0.2978	0.8330	0.9789	-
5.0	0.0035	<0.0001	0.0001	0.0002
**DISAGGREGATED CELL DENSITY**				
**Pores - One-way ANOVA:** p=0.0010				
**Tukey's Multiple Comparisons Tests**				
Intensity (W/cm^2)	0.5 (300 s)	0.5 (350 s)	2.0	
0.5 (350 s)	0.9437	-	-	
2.0	0.7782	0.9764	-	
5.0	0.0013	0.0024	0.0039	
**Surface – One-way ANOVA:** p<0.0001				
**Tukey's Multiple Comparisons Tests**				
Intensity (W/cm^2)	Control	0.5 (300 s)	0.5 (350 s)	2.0
0.5 (300 s)	0.0004	-	-	-
0.5 (350 s)	0.0004	>0.9999	-	-
2.0	0.0007	0.9828	0.9920	-
5.0	0.9599	0.0008	0.0010	0.0017
